# Frequency of Hyponatremia and Its Impact on Prognosis in Ischemic Stroke

**DOI:** 10.7759/cureus.40317

**Published:** 2023-06-12

**Authors:** Asif Khan, Zamin Khan, Salman Khan, Assam Ullah, Gohar Ayub, Muhammad Naveed Tariq

**Affiliations:** 1 Department of Medicine, Khyber Teaching Hospital, Peshawar, PAK

**Keywords:** nihss, mortality, outcome, prognosis, ischemic stroke, hyponatremia

## Abstract

Background

Hyponatremia, often encountered in hospitalized patients, is associated with adverse outcomes in ischemic stroke patients. In this study, we investigated the frequency of hyponatremia and its impact on prognosis and clinical outcomes in ischemic stroke patients from a tertiary care hospital.

Methodology

A total of 289 patients admitted to the hospital with ischemic stroke from September 2022 to February 2023 were considered in this cross-sectional study. Serum sodium level was measured on admission, and hyponatremia was defined as sodium less than 135 mmol/L. The primary outcome of the study was assessed by the National Institutes of Health Stroke Scale (NIHSS) score on admission and discharge and inpatient mortality. Data were analyzed using SPSS version 20 (IBM Corp., Armonk, NY, USA), and multivariate logistic regressions were conducted using variables identified as having a relationship with hyponatremia.

Results

Our study shows that among 289 patients with ischemic stroke, the mean age was 61 ± 8.53 years. Hyponatremia was observed in 101 (35%) patients, and all baseline characteristics and risk factors for stroke were similar between patients with and without hyponatremia. The patients with hyponatremia had higher NIHSS scores on admission (p = 0.041) and at discharge (p = 0.039). In the resultant multivariate analysis, hyponatremia was an independent predictor of mortality rates during the hospital stay. The cumulative incidence rates of in-hospital mortality for hyponatremia and normal sodium level were 16.8% and 10.1%, respectively.

Conclusions

Hyponatremia is prevalent in ischemic stroke and is independently associated with in-hospital mortality and worse NIHSS scores at admission and discharge.

## Introduction

Despite recent advances in medicine, stroke is the second-leading cause of death and the third-leading cause of death and disability worldwide [1]. Ischemic stroke, defined as an episode of neurological dysfunction caused by focal cerebral, spinal, or retinal infarction, comprises 65% of all strokes [2]. Electrolyte disturbances are usually secondary to comorbidities associated with strokes, such as diabetes mellitus, hypertension, and congestive cardiac failure.

Hyponatremia (serum sodium <135 mmol/L), one of the most common electrolyte abnormalities encountered in hospitals, is frequently encountered in ischemic stroke patients [3,4]. Hyponatremia is commonly reported in subarachnoid hemorrhage, ischemic stroke, and intracerebral hemorrhage [5,6]. Hyponatremia in the setting of an acute stroke may adversely impact the course of the disease, mainly by increasing brain edema and subsequent neurological consequences [6]. Although hyponatremia is usually attributed to the comorbidities related to stroke, the proposed mechanisms of hyponatremia caused by stroke are cerebral salt wasting (CSW), syndrome of inappropriate antidiuretic hormone (SIADH), and pituitary ischemia [6,7]. A meta-analysis including 21,973 stroke cases established that hyponatremia was associated with a higher risk of all-cause mortality in the short-term (hazard ratio = 1.78, 95% confidence interval (CI) = 1.19-2.75) and long-term follow-up (hazard ratio = 2.23, 95% CI = 1.30-3.82) [8]. In a study by Rodrigues et al., worse National Institute of Health Stroke Scale (NIHSS) scores at admission as well as at discharge were observed in the hyponatremia group patients compared with patients with normal sodium levels [9]. Consequently, prompt recognition and management of hyponatremia in stroke patients may decrease poor outcomes and deaths.

In this study, we aimed to assess the frequency of hyponatremia and a possible association between hyponatremia and the prognosis of stroke in terms of severity and in-hospital mortality.

## Materials and methods

This cross-sectional, descriptive study was approved by the Institutional Research and Ethical Review Board of Khyber Medical College Peshawar, Pakistan on September 01, 2022. All consecutive ischemic stroke patients, per the American Heart Association/American Stroke Association definition, admitted to the Department of Medicine, Khyber Teaching Hospital during a six-month period (September 01, 2022, to February 28, 2023) were reviewed [10]. All patients with hemorrhagic stroke were excluded from the review. All patients provided written informed consent. In certain cases where patients experienced severe motor and cognitive impairments, consent was obtained from their relatives or caregivers. Serum sodium level was measured on admission, and hyponatremia was defined as serum sodium of less than 135 mmol/L [11]. In our laboratory, the measurement of sodium level is made using the direct ion-selective electrode method, so the possibility of pseudo-hyponatremia is immediately excluded. The primary outcome of the study was assessed by the NIHSS score at discharge and inpatient mortality. The characteristics of patients recorded were age, gender, any history of comorbidities, and status of hyponatremia.

Data were entered and statistical analyses were performed using SPSS version 20.0 (IBM Corp., Armonk, NY, USA). Mean and standard deviation (SD) was calculated for numerical variables such as age. Frequency and percentage were calculated for categorical variables such as gender, history of comorbidities, and status of hyponatremia. Effect modifiers such as age, gender, and history of comorbidities were controlled through stratification. Post-stratification, the chi-square test was applied and multivariate logistic regressions were conducted using variables identified as having a relationship with hyponatremia, including age, cigarette smoking status, history of hypertension, history of diabetes mellitus, history of coronary heart disease, history of atrial fibrillation, hyperlipidemia, admission NIHSS score, and malignancy. A p-value ≤0.05 was taken as significant at a 95% confidence interval (CI).

## Results

In this study, 289 patients were diagnosed with ischemic stroke, and serum sodium levels were measured on admission. Hyponatremia was observed in 101 (35%) patients. Among the 289 patients analyzed, 78 (27%) patients were in the age range 18-40 years, and 211 (73%) patients were in the age range 41-75 years. The mean age was 61 years with an SD of ±7.93. Gender distribution among the 289 patients analyzed was 162 (56%) patients male while 127 (44%) patients were female. All baseline characteristics and risk factors for stroke were similar between patients with and without hyponatremia (Table 1).

**Table 1:** Baseline characteristics between patients with hyponatremia and without hyponatremia.

Characteristics	Hyponatremia (%)	No hyponatremia (%)	P-value
Age	63.4	60.6	0.52
Gender			0.14
Male	52 (32.1%)	110 (67.9%)	
Female	49 (38.6%)	78 (62.4%)	
Hypertension	61 (60.3%)	99 (52.6%)	0.08
Diabetes mellitus	62 (61.3%)	101 (53.7%)	0.11
Smoking	46 (45.5%)	78 (41.4%)	0.54
Hyperlipidemia	32 (31.6%)	58 (30.8%)	0.09
Ischemic heart disease	27 (26.7%)	45 (23.9%)	0.23
Malignancy	8 (7.9%)	16 (8.5%)	0.35
Obesity	59 (58.4%)	98 (52.1%)	0.09
Atrial fibrillation	14 (13.8%)	20 (10.6%)	0.56

Compared to participants with normal serum sodium levels, individuals with hyponatremia were more likely to be older and experienced a more severe stroke, as indicated by their NIHSS scores. NIHSS score in the severe score range at admission was observed in 19.8% of the hyponatremia group compared with 15.9% in the patients with normal sodium levels (p = 0.041). Furthermore, patients with hyponatremia had higher rates of in-hospital mortality compared to patients with normal sodium levels (p = 0.026). Additionally, a smaller proportion of patients in the hyponatremia group were discharged to their homes compared to those with normal sodium levels. Patients with hyponatremia exhibited higher NIHSS scores at the time of discharge compared to those with normal sodium levels. In patients with normal sodium levels, 14.9% had an NIHSS score in the severe score range, whereas, in patients with hyponatremia, 16.8% had a severe score. This reached a statistical significance (p = 0.039) (Table 2).

**Table 2:** Functional status on admission and at discharge and in-hospital mortality after ischemic stroke in patients with and without hyponatremia. NIHSS: National Institute of Health Stroke Scale

Parameter	Hyponatremia (%)	No hyponatremia (%)	P-value
NIHSS on admission			0.041
1–4	23 (22.7%)	55 (29.3%)	
5–15	31 (30.6%)	56 (29.8%)	
16–20	27 (26.7%)	47 (25.0%)	
21–42	20 (19.8%)	30 (15.9%)	
NIHSS at discharge			0.039
1–4	35 (34.7%)	80 (42.5%)	
5–15	29 (28.7%)	50 (26.6%)	
16–20	20 (19.8%)	30 (15.9%)	
21–42	17 (16.8%)	28 (14.9%)	
In-hospital mortality	17 (16.8%)	19 (10.1%)	0.026

In the resultant multivariate analysis, hyponatremia was an independent predictor of mortality rates during the hospital stay and worse NIHSS scores at discharge. In this analysis, age (odds ratio (OR) = 1.31, p = 0.027), hypertension (OR = 1.39, p = 0.021), history of ischemic heart disease (OR = 1.42, p = 0.042), atrial fibrillation (OR = 2.12, p = 0.009), hyponatremia (OR = 1.67, p = 0.041), and baseline NIHSS score (OR = 3.70, p = 0.008) were significantly associated with all-cause in-hospital mortality (Table 3). In relation to NIHSS score at discharge (Table 4), significant associations were observed between age (OR = 1.79, p = 0.018), diabetes mellitus (OR = 1.28, p = 0.045), hypertension (OR = 1.54, p = 0.019), history of ischemic heart disease (OR = 1.49, p = 0.049), atrial fibrillation (OR = 1.72, p = 0.018), serum sodium (OR = 1.87, p = 0.030), and baseline NIHSS score (OR = 2.90, p = 0.011). The cumulative incidence rates of in-hospital mortality for hyponatremia and normal sodium level were 16.8% and 10.1%, respectively.

**Table 3:** Association between baseline characteristics and in-hospital mortality in patients with ischemic stroke in multivariate analyses. IHD: ischemic heart disease; OR: odds ratio; CI: confidence interval

Variable	OR (95% CI)	P-value
Age	1.31 (1.08-1.54)	0.027
Cigarette smoking	1.19 (0.96-1.42)	0.323
Diabetes mellitus	0.89 (0.66-1.12)	0.701
Hypertension	1.39 (1.16-1.62)	0.021
IHD	1.42 (1.19-1.65)	0.042
Atrial fibrillation	2.12 (1.89-2.35)	0.009
Hyponatremia	1.67 (1.44-1.90)	0.041
NIHSS on admission	3.70 (3.47-3.93)	0.008
Hyperlipidemia	1.12 (0.89-1.35)	0.604
Malignancy	1.01 (0.78-1.24)	0.190

**Table 4:** Association between baseline characteristics and NIHSS score at discharge in patients with ischemic stroke in multivariate analyses. IHD: ischemic heart disease; OR: odds ratio; CI: confidence interval

Variable	OR (95% CI)	P-value
Age	1.79 (1.56-2.02)	0.018
Cigarette smoking	1.23 (0.99-1.46)	0.134
Diabetes mellitus	1.28 (1.05-1.51)	0.045
Hypertension	1.54 (1.31-1.77)	0.019
IHD	1.49 (1.26-1.72)	0.049
Atrial fibrillation	1.72 (1.49-1.95)	0.018
Hyponatremia	1.87 (1.64-2.10)	0.030
NIHSS on admission	2.90 (2.67-3.13)	0.011
Hyperlipidemia	1.22 (0.99-1.45)	0.124
Malignancy	1.07 (0.84-1.30)	0.334

## Discussion

Hyponatremia is a common electrolyte abnormality often coexisting with stroke and is related to the clinical outcome in these patients. It has been reported that hyponatremia is an independent predictor of mortality and poor outcome due to worsening cerebral edema, which might lead to neurological consequences and death [12]. Our study evaluates the frequency of hyponatremia and its value as a prognostic factor for clinical outcomes and mortality in patients with acute ischemic stroke.

The present study of 289 patients with a mean age of 61 (SD ±7.93) years demonstrated the presence of hyponatremia in 101 (35%) patients. Among the 101 patients, 51.5% were male and 48.5% were female. A prospective study conducted in 2014 showed a similar incidence of hyponatremia (35.3%) [13]. Unlike our study, this study also included patients with hemorrhagic stroke. Rodrigues et al. conducted a study on ischemic stroke patients and hyponatremia was observed in 16% of patients [9]. Although the exact mechanism remains a matter of debate, the two widely proposed potential causes of hyponatremia in stroke are SIADH and CSW [7,14]. Interestingly, many studies have demonstrated the relation of NIHSS score with hyponatremia on admission, during admission, and at discharge. Rodriques et al. claimed hyponatremia as an independent prognostic factor associated with poor NIHSS score on admission (p = 0.032) and at discharge (p = 0.02) after ischemic stroke and poor discharge disposition (p = 0.004) [9]. In our study, NIHSS scores were used to predict disability after stroke, and patients with hyponatremia were found to have worse NIHSS scores both at admission (p = 0.041) and discharge (p = 0.039). The possible underlying mechanism of increased disability and poor outcomes in hyponatremic patients after ischemic stroke may be attributed to the subsequent worsening of cerebral edema associated with ischemic injury and the resultant osmotic demyelination due to aggressive correction of hyponatremia [12,15]. Hyponatremia has also been associated with acute confusion, nausea, vomiting, seizures, and, in severe cases, coma, followed by death due to brain herniation [16]. A recent study in 2022 demonstrated that lower serum sodium levels were independently associated with a poor three-month outcome (OR = 1.647; 95% CI = 1.012-2.679) in ischemic stroke patients [17].

In the present study of ischemic stroke patients, hyponatremia at admission was independently associated with in-hospital mortality accounting for confounders, including age, baseline NIHSS score, and comorbidities such as hypertension and atrial fibrillation. Huang et al. reported that hyponatremia was a significant predictor of three-year mortality in patients with first-ever ischemic stroke after adjustment for related variables (HR = 2.23, 95% CI = 1.30-3.82, p = 0.003). A separate study conducted in 2019 revealed a significant association between hyponatremia upon admission in patients with ischemic stroke and short-term mortality, which was defined as either death during the initial hospital stay or a composite outcome of in-hospital mortality (p = 0.026) [18]. Furthermore, in a study conducted by Soiza et al., hyponatremia found at admission in acute stroke (both ischemic and hemorrhagic) patients was independently associated with higher mortality in patients below 75 years of age [19]. Contrary to what has been reported in most studies and our study, a multicentric study conducted in China found no significant association between hyponatremia and in-hospital mortality after adjusting for possible confounding factors (p = 0.905) [20]. Therefore, this is still a matter of debate whether hyponatremia independently predicts mortality in stroke patients or whether it is merely a bystander affecting a hospitalized patient. Consequently, regular assessment of serum sodium concentration during treatment is crucial to determine the effectiveness of hyponatremia treatment in patients with acute ischemic stroke and to prevent any improper increase in serum sodium levels (i.e., >10-12 mmol/L/day) due to the risk of central pontine myelinolysis [6,21,22]. Taken together, whether correcting hyponatremia in patients with acute stroke would improve clinical outcomes or decrease mortality still remains unclear and needs to be answered in future studies.

Our study does not provide any insight into the etiology and mechanism of hyponatremia, or how can we treat it. It is one of the limitations of the study. Our study also has some other shortcomings. As it was a single-center observational study, causality cannot be established, though it is an important addition to the current evidence. We only used the sodium levels measured at admission to assess the role of hyponatremia on disability and mortality in ischemic stroke. We did not take into account the severity of hyponatremia (mild, moderate, severe) while determining its impact on the outcome. Lastly, we only observed in-hospital mortality and did not follow the patients after discharge to establish the role of hyponatremia in short-term and long-term mortality after ischemic stroke.

## Conclusions

Hyponatremia is commonly observed in acute ischemic stroke. This study shows that hyponatremia is significantly associated with poor NIHSS scores on admission and at discharge in acute ischemic stroke patients. The results of the study after multivariate analysis also suggest that hyponatremia is an independent prognostic factor for in-hospital mortality and disability at discharge in these patients. In brief, the independent association between hyponatremia at admission and disability as well as in-hospital mortality in ischemic stroke highlights the importance of monitoring and therapeutically managing sodium levels, which could potentially enhance prognosis.
